# Abdominal aortic calcification is superior to other arteries calcification in predicting the mortality in peritoneal dialysis patients – a 8 years cohort study

**DOI:** 10.1186/s12882-019-1593-6

**Published:** 2019-12-02

**Authors:** Qingyu Niu, Huiping Zhao, Bei Wu, Shihming Tsai, Jian Wu, Meng Zhang, Lixia Lu, Jie Qiao, Chuncui Men, Li Zuo, Mei Wang

**Affiliations:** 1 Department of Nephrology, Peking University People’s Hospital, Beijing, China; 2 Department of Nephrology, Beijing Tsinghua Changgung Hospital, Beijing, China; 3 Department of Radiology, Peking University People’s Hospital, Beijing, China

**Keywords:** Cardiovascular, Peritoneal Dialysis, Mortality, Outcomes, Vascular calcification

## Abstract

**Background:**

In recent years, there has been a growing concern that abdominal aortic calcification (AAC) has a predictive effect on the prognosis of patients with end-stage renal disease (ESRD). However, whether other vascular calcification (VC) can predict the occurrence of adverse events in patients, and whether it is necessary to assess the calcification of other blood vessels remains controversial. This study aimed to assess VC in different sites using X-ray films, and to investigate the predictive effects of VC at different sites on all-cause mortality and cardiovascular (CV) mortality in peritoneal dialysis (PD) patients.

**Methods:**

The data of Radiographs (lateral abdominal plain film, frontal pelvic radiograph and both hands radiograph) were collected to evaluate the calcification of abdominal aorta, iliac artery, femoral artery, radial artery, and finger arteries. Patients’ demographic data, clinical characteristics, laboratory data were recorded. The total follow-up period was 8 years, and the time and cause of death were recorded. Survival curves were estimated using Kaplan-Meier analysis. COX regression analysis was used to examine independent predictors of all-cause mortality and CV mortality.

**Results:**

One hundred fifty PD patients were included, a total of 79 patients (52.7%) died at the end of follow-up. After adjusting variables in the multivariate COX regression analysis, AAC was an independent predictor of all-cause mortality in PD patients (HR = 2.089, 95% CI: 1.089–4.042, *P* = 0.029), and was also an independent predictor of CV mortality (HR = 4.660, 95% CI: 1.852–11.725, *P* = 0.001). We also found that femoral artery calcification had a predictive effect on all-cause and CV mortality. But the calcification in iliac artery, radial artery, and finger arteries were not independent predictors of patients’ all-cause and CV mortality in PD patients.

**Conclusion:**

AAC was more common in PD patients and was an independent predictor of all-cause mortality and CV mortality. The femoral artery calcification also can predict the mortality, but the calcification of iliac artery, radial artery, and finger arteries cannot predict the mortality of PD patients.

## Introduction

Vascular calcification (VC) is a common complication in patients with end stage renal disease (ESRD), and it is one of the most common causes of cardiovascular disease (CVD) [1, 2]. In ESRD patients, the incidence of CVD is 20 to 30 times higher than that of the general population [3], and cardiovascular death is the leading cause of death in dialysis patients [4, 5]. The clinical practice guideline for chronic kidney disease-mineral and bone disorder (CKD-MBD) of Kidney Disease: Improving Global Outcomes (KDIGO) suggested that if patients with chronic kidney disease (CKD) stage 3–5 have vascular or valvular calcification, they should be seen as having highest CV risk, and use this information to guide the management of CKD-MBD [6, 7].

Previous studies investigated the relationship between VC or valvular calcification and the prognosis of patients with ESRD. Studies showed that in patients with hemodialysis (HD) and PD treatment, aortic arch calcification is an independent predictor of CV mortality and all-cause mortality. As the degree of calcification increases, the risk of poor prognosis increases [8–11]. Von et al. [12] found that high coronary calcification score is an independent risk factor for CV mortality and non-CV mortality in HD patients. In addition, several studies suggested that the presence and severity of valvular calcification is associated with an increased risk of all-cause mortality in HD patients [13, 14]. Take all the above together, it can be concluded that it is necessary to assess VC for patients on HD or PD. 

In daily clinical work, it is necessary to assess VC of patients. There are several different methods for assessing VC: electron beam computed tomography (EBCT) or multislice computed tomography (MSCT) for coronary artery assessment, abdominal aortic calcification (AAC) score varying from 0 to 24 points developed by Kauppila et al. [15], aortic calcification score ranged from 0 to 8 points developed by Schousboe et al. [16], AAC index expressed as a percentage [17, 18], thoracic aortic calcification index, calcification score of iliac, femoral, radial and finger arteries varying from 0 to 8 points developed by Adragao et al. [19], assessing calcification of iliac artery by pelvic CT, and assessing cardiac valve calcification by echocardiography and so on. Using EBCT and MSCT to assess VC in patients is expensive and unsuitable for widely use in dialysis centers. The KDIGO guideline recommended that for patients with CKD stage 3-5D, lateral abdomen X-ray can be used to detect the presence of vascular calcification, which is a reasonable alternative to CT [20].

Although the prevalence of VC is higher in patients with CKD, arteries in some areas are not sensitive to calcification comparing with main arteries, such as finger arteries [21]. O’Neill et al. performed histological studies on arterioles with a diameter of 10-1500um, suggesting that the mechanism of calcification in different types and sizes of arteries may be different [22]. Adragao et al. [19] indicated that the calcification score of small and medium arteries has predictive value for the occurrence of CVD in patients. In our previous study, we found that the influencing factors were different in VCs occurred in different sites in peritoneal dialysis (PD) patients [23].

At present, there were few studies focused on the predictive effect of calcification of iliac artery, femoral artery, radial artery and finger arteries on the prognosis of dialysis patients, especially PD patients. Whether it is necessary to assess the calcification of arteries in different sites remains controversial. Therefore, we conducted a retrospective cohort study, using X-ray films to assess VC of different sites, and to investigate the relationship between VC and all-cause mortality and CV mortality in PD patients.

## Materials and methods

### Study design and subjects

This study enrolled maintenance PD patients with ESRD in Peking University People’s Hospital PD center. Inclusion criteria for participants were age > 18 years old, PD duration ≥6 months, in stable clinical condition and regular follow-up. Exclusion criteria: patients transformed from HD or received kidney transplantation; patients withdrew during the follow-up period (transfer to hemodialysis, receive kidney transplantation or move to other center). Radiographs (lateral abdominal radiograph, frontal pelvic radiograph and both hands radiograph) were obtained for each patient between April 2010 and December 2014. Patients were followed up until May 2018. The death time and cause were recorded in death cases. All follow-up information was obtained from our Center’s medical records. A total of 154 patients met the criteria, but 4 patients (one for lacking laboratory examination data, and three for without radiographs) were excluded. So a total of 150 patients were included in this study. The primary end point was death due to any cause (all-cause death). The secondary end point was CV death, defined as death due to heart failure, myocardial infarction, arrhythmia, sudden cardiac death, cerebrovascular disease, atherosclerotic heart disease and cardiomyopathy.

The study was approved by the Ethics Committee of Peking University People’s Hospital (ethical approval number: 2018PHB149). As this study was a retrospective observational cohort study without any intervention, informed consent was exempted by the Ethics Committee.

### Demographic and clinical data

Demographic and clinical characteristics were collected, including age, gender, PD duration, primary disease, the history of diabetes, hypertention, CVD, cerebrovascular dialysate glucose load, urinary output and body mass index (BMI). laboratory indices including serum total calcium (Ca), phosphate (P), serum intact parathyroid hormone (iPTH), alkaline phosphatase (ALP), albumin (ALB), triglycerides (TG), low-density lipoprotein cholesterol (LDL-C), carbon dioxide combining power (CO_2_CP), hemoglobin (HB) and total urea clearance (Kt/V). Mean values of 3 measurements during the 3 months before radiographs. The mean values of systolic blood pressure (BP), diastolic BP, pulse pressures and urinary output during the 3 months before X-ray examination were calculated.

### Evaluation the vascular calcification of different sites

We used lateral abdominal radiograph, frontal pelvic radiograph and both hands radiograph to evaluate the calcification of abdominal aorta, iliac artery, femoral artery, radial artery and finger arteries. This method was described by WANG Mi and WANG Mei et al. previously [24–26], and it was an improvement based on the method described by Adragao et al. [19]. In Adragao’s method, radiographs of the pelvis were divided by 2 lines: a horizontal line just above the femoral heads and a median vertical line; and radiographs of each hand were divided by a horizontal lone over the proximal end of the metacarpals, with total 8 points. The method of WANG Mi and WANG Mei was also added the score of abdominal aorta: the lateral abdominal radiographs were divided into two sections by a horizontal line over the intervertebral space between L2 and L3, and the presence of calcification was given 1 point for each part. Scores from all parts summed up to a total score, which ranging from 0 to 10. The radiographs were reviewed by 2 radiologists blindly. For the inconsistent results, 2 radiologists re-scored and discussed together and then gave a unified result.

Meanwhile, to investigate the effect of severity of VC on patients’ prognosis, patients were divided into 4 groups according to VC score: 0 point was classified as no calcification, 1 to 3 points was classified as mild calcification, and 4 to 6 points was classified as moderate calcification, > 6 points was classified as severe calcification.

### Statistics

Continuous variables were expressed as mean ± standard deviation or median with IQR, and categorical data were expressed as number and percentages. Differences in mean and median values between groups with and without AAC were evaluated by using independent sample t-test or Wilcoxon rank-sum test respectively. Categorical data between groups were compared by using chi-square test. Survival curves were estimated by the Kaplan-Meier method and evaluated using log-rank test. Survival curves after adjusting variables were estimated by multivariate COX regression analysis. Variables were examined first by using the univariate Cox regression analysis, and significant variables were forced into multivariate Cox regression analysis. Age, TG, and BMI were included in the univariate and multivariate COX regression analysis as categorical variables. Among them, Age were divided into two groups by < 65 years old and ≥ 65 years old. TG was divided into two groups as normal group (< 1.70 mmol/L) and high (≥1.70 mmol/L) group. BMI was divided into low (< 18.50 kg/m^2^), normal (18.50–23.99 kg/m^2^) and high (≥24 kg/m^2^) three groups. The effect size is expressed in the form of Hazard ratio (HR) and its 95% confidence intervals (CI). *P* value < 0.05 was considered to be statistically significant. Statistical analysis was performed using SPSS software, version 22.0(IBM Corp., Armonk, NY, USA).

## Results

### Demographic data and clinical characteristics

A total of 150 PD patients were included, including 76 males (50.7%), with an average age of 60.4 ± 14.0 years (21–75) and a median dialysis vintage of 24 (16, 39) months. Primary renal diseases were predominantly diabetic nephropathy (*n* = 59, 39.3%), followed by chronic glomerulonephritis (*n* = 57, 38.0%), chronic tubulointerstitial nephropathy (*n* = 15, 10.0%), hypertensive renal disease (*n* = 17, 11.3%), and others (*n* = 2, 1.3%). There were 64 patients (42.7%) with diabetes. 37 patients (24.7%) with CVD history and 17 (11.3%) with cerebrovascular disease history (Table 1). Among these patients, 136 patients (90.7%) were treated with CAPD, 9 (6.0%) were treated with IPD and 5 (3.3%) were treated with APD. All the patients used conventional glucose-based, lactate-buffered PD solutions (Ultrabag; Baxter Healthcare, Guangzhou, China; Mg2+ 0.25 mmol/L, Ca2+ 1.25 mmol/L or 1.75 mmol/L, Na+ 132 mmol/L, and Cl– 96 mmol/L). The daily dialysate exchange dose was more than 6 L, received either by continuous ambulatory PD (CAPD) or intermittent PD.

**Table 1:** Baseline characteristics of patients according to the presence or absence of abdominal aortic calcification

Variables	All (*n* = 150)	Presence of AAC (*n* = 91)	Absence of AAC (*n* = 59)	*P*
Age (years)	60.37 ± 13.98	65.86 ± 11.47	51.92 ± 11.33	< 0.001
Age (n, %)				< 0.001
< 65	87 (58.00)	37 (40.66)	50 (84.75)	
≥ 65	63 (42.00)	54 (59.34)	9 (15.25)	
Gender (n, %)				0.765
Female	74 (49.33)	44 (48.35)	30 (50.85)	
Male	76 (50.67)	47 (51.65)	29 (49.15)	
Cardiovascular disease (n, %)	37 (24.67)	19 (12.67)	18 (12.00)	0.181
Cerebrovascular disease (n, %)	17 (11.33)	12 (8.00)	5 (3.33)	0.374
Use of Vitamin D (n, %)	54 (36.00)	29 (31.87)	25 (42.37)	0.224
Hypertention (n, %)	125 (83.33)	81 (54.00)	44 (29.33)	0.012
Diabetes (n, %)	64 (42.67)	50 (33.33)	14 (9.33)	< 0.001
PD vintage (months)	24 (16,39)	30 (17,43)	20 (5,31)	0.015
BMI (kg/m^2^)	23.04 ± 3.74	23.53 ± 3.75	22.27 ± 3.21	0.043
BMI (n, %)				0.073
< 18.50	14 (9.33)	6 (6.59)	8 (13.56)	
18.50–23.99	80 (53.34)	45 (49.45)	35 (59.32)	
≥ 24.00	56 (37.33)	40 (43.96)	16 (27.12)	
SBP (mmHg)	129.48 ± 17.60	127.36 ± 13.94	130.85 ± 19.57	0.236
DBP (mmHg)	77.66 ± 11.57	74.65 ± 10.72	82.32 ± 11.37	< 0.001
Pulse pressure (mmHg)	51.81 ± 16.36	56.21 ± 17.68	45.03 ± 11.21	< 0.001
Urinary output (ml/day)	350 (100,753)	300 (100,600)	600 (200,900)	0.004
Dialysate glucose load (g/day)	156.34 ± 40.62	162.64 ± 42.20	146.62 ± 36.29	0.021
Dialysate calcium load (g/day)	0.47 ± 0.10	0.46 ± 0.11	0.47 ± 0.09	0.233
Total Kt/V(/week)	1.88 (1.62,2.12)	1.93 (1.71,2.21)	1.80 (1.58,2.11)	0.825
nPCR (g/d)	1.12 ± 0.25	1.10 ± 0.26	1.14 ± 0.23	0.334
HB (g/L)	114.53 ± 8.13	113.66 ± 7.97	115.88 ± 8.25	0.102
ALB (g/L)	38.77 ± 3.22	38.11 ± 3.00	39.78 ± 3.31	0.002
Ferritin (ng/ml)	588.8 (374.1,796.3)	649.3 (386.4,813.6)	528.3 (342.7,787.8)	0.165
Ca (mmol/L)	2.32 ± 0.27	2.32 ± 0.29	2.31 ± 0.25	0.941
P (mmol/L)	1.49 ± 0.32	1.52 ± 0.34	1.43 ± 0.29	0.100
iPTH (pg/ml)	140.20 (64.00,266.63)	140.40 (53.90,287.00)	140.00 (93.10,250.90)	0.570
ALP (U/L)	72.84 (57.88,92.25)	74.50 (60.50,97.00)	72.00 (56.00,90.00)	0.143
CO_2_ (mmol/L)	27.53 ± 2.61	27.19 ± 2.90	28.04 ± 2.00	0.052
TG (mmol/L)	1.82 (1.33,2.50)	1.92 (1.47,2.84)	1.54 (1.15,2.15)	0.002
TG (n, %)				0.012
< 1.70	65 (43.33)	32 (35.16)	33 (55.93)	
≥ 1.70	85 (56.67)	59 (64.84)	26 (44.07)	
LDL-C (mmol/L)	2.85 ± 0.75	2.86 ± 0.79	2.83 ± 0.69	0.837
All-cause deaths	79 (52.67%)	64 (42.67)	15 (10.00)	< 0.001
Cardiovascular deaths	36 (24.00)	30 (20.00)	6 (4.00)	< 0.001
Modality of dialysis (n, %)				0.355
CAPD	136 (90.67)	82 (90.11)	54 (91.53)	
IPD	9 (6.00)	7 (7.69)	2 (3.39)	
APD	5 (3.33)	2 (2.20)	3 (5.08)	

*BMI* Body mass index, *SBP* Systolic blood pressure, *DBP* Diastolic blood pressure, *nPCR* Normalized protein catabolic rate, *HB* Hemoglobin, *ALB* Albumin, *Ca* Calcium, *P* Phosphate, *iPTH* Intact parathyroid hormone, *ALP* Alkaline phosphatase, *TG* Triglyceride, *LDL-C* Low density lipoprotein cholesterol, *CAPD* Continuous ambulatory peritoneal dialysis, *IPD* Intermittent peritoneal dialysis, *APD* Automated peritoneal dialysis

Median follow-up duration was 44.5 (range 2 to 96) months. At the end of follow-up, only 46 patients were still on PD treatment. During follow-up, 6 patients received kidney transplant, 17 were switched to hemodialysis, and 24 lost their follow-up (these patients were excluded in statistic analysis). Seventy-nine patients (52.7%) died. A CV death was reported in 36 patients (56%), and the mainly cause of CV death including: myocardial infarction (9), sudden cardiac death (17), cerebrovascular disease (9) and heart failure (1). Furthermore, there were 43 non-CV deaths, including infections (24), tumors (5), gastrointestinal bleeding (2), and others (12).

### Abdominal aortic calcification

91 (60.7%) of the patients had AAC. Compared with patients without AAC, patients with AAC had older age, higher proportion of hypertension and diabetes, longer dialysis duration, higher BMI and TG levels, and higher dialysate glucose load, lower diastolic blood pressure (DBP), less urinary output, and lower ALB level. See Table 1.

**Fig. 1:**
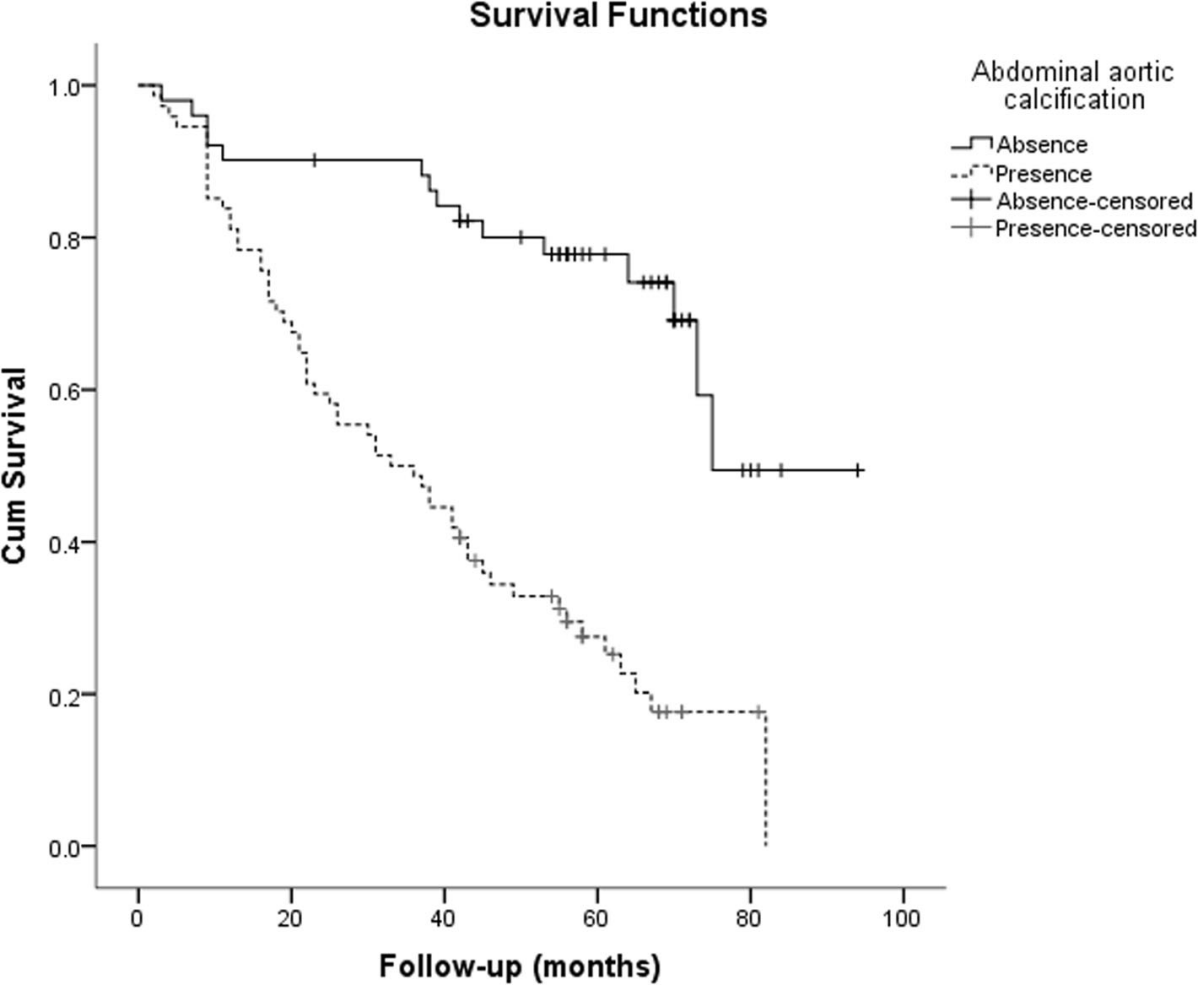
The Kaplan-Meier curve of All death mortality of 150 peritoneal dialysis patients. Patients with abdominal aortic calcification showed significantly greater death from all causes than those without; log-rank test, *P* < 0.001

**Fig. 2:**
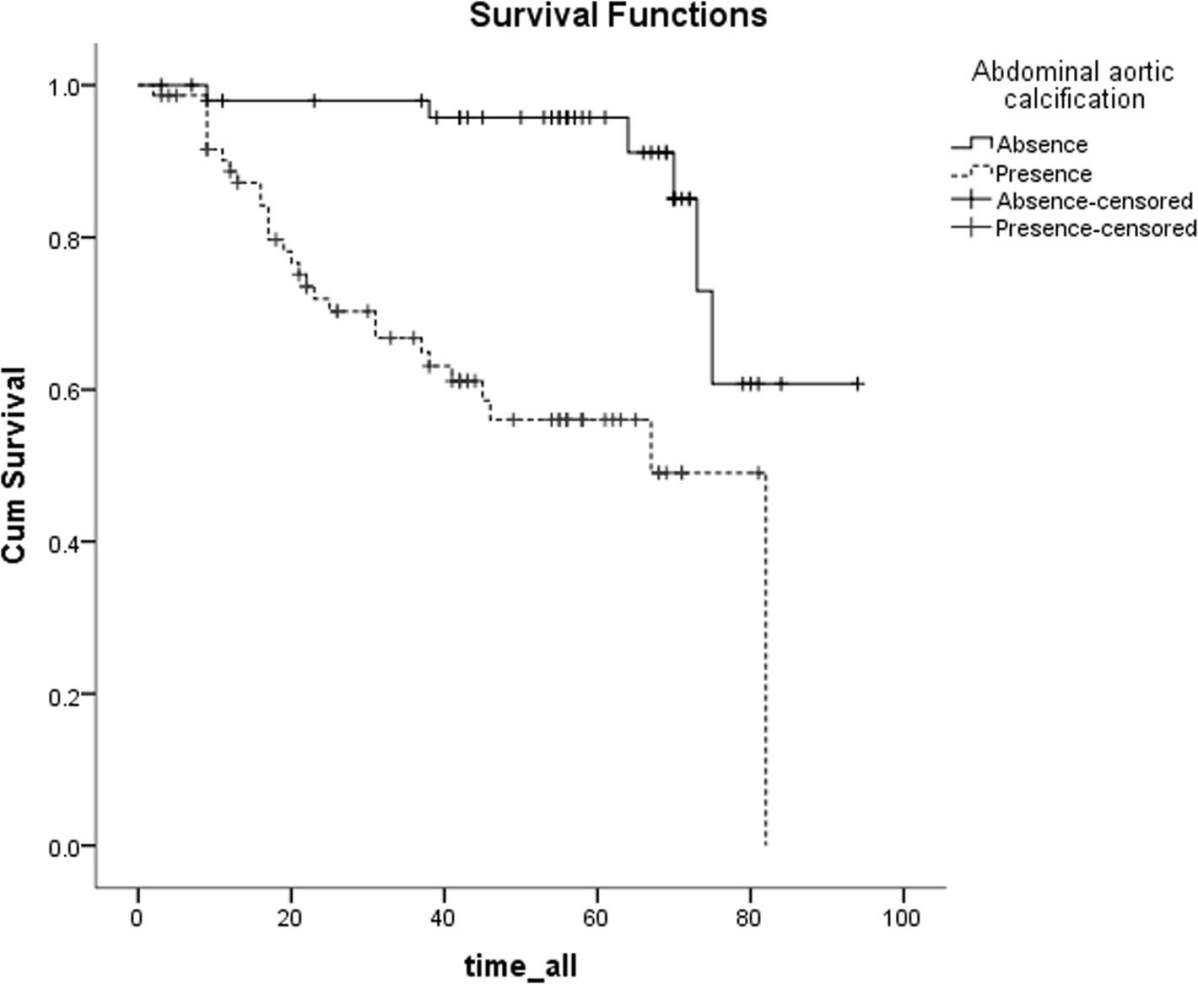
The Kaplan-Meier curve of cardiovascular mortality of 150 peritoneal dialysis patients. Patients with abdominal aortic calcification showed significantly greater cardiovascular death than those without; log-rank test, *P* < 0.001

Kaplan-Meier analysis was used to examine the relationship between AAC and patients’ prognosis. Compared with patients without AAC, the risk of all-cause mortality was significantly higher in patients with AAC (log-rank test, *P* < 0.001, Fig. 1), and the risk of CV mortality was also significantly higher (log-rank test, *P* < 0.001, Fig. 2). In the univariate COX regression analysis, age ≥ 65 years, history of hypertension, diabetes, BMI ≥ 24.00, lower DBP, lower ALB, TG ≥1.7 mmol/L, higher LDL-C, HCO3- and AAC were predicted indicators of all-cause mortality of patients. Age ≥ 65 years, diabetes, lower ALB, higher LDL-C, history of cerebrovascular disease and AAC were predictors of CV mortality (Table 2).

**Table 2:** Univariate Cox proportional hazards analysis of factors associated with all-cause and cardiovascular mortality in peritoneal dialysis patients

Variable	All-cause mortality		Cardiovascular mortality	
	HR (95% CI)	*P*	HR (95% CI)	*P*
Age (years)				
< 65	Ref.		Ref.	
≥ 65	5.491 (3.262–9.245)	< 0.001	3.845 (1.879–7.869)	< 0.001
Gender (male vs. female)	1.370 (0.860–2.180)	0.185	1.435 (0.735–2.803)	0.290
Cardiovascular disease (yes vs. no)	0.957 (0.564–1.622)	0.869	1.020 (0.485–2.147)	0.958
Cerebrovascular disease (yes vs. no)	1.582 (0.783–3.197)	0.201	3.122 (1.404–6.943)	0.005
Hypertension (yes vs. no)	2.191 (1.048–4.580)	0.037	3.012 (0.919–9.870)	0.069
Diabetes (yes vs. no)	2.806 (1.737–4.533)	< 0.001	4.149 (1.981–8.689)	< 0.001
PD vintage (/1 month)	1.001 (0.992–1.011)	0.789	0.988 (0.969–1.009)	0.258
BMI (kg/m^2^)				
< 18.50	Ref.		Ref.	
18.50–23.99	2.191 (0.779–6.162)	0.137	3.492 (0.460–26.491)	0.226
≥ 24.00	2.852 (1.004–8.101)	0.049	6.220 (0.827–46.777)	0.076
SBP (/1 mmHg)	1.011 (0.997–1.024)	0.120	1.020 (1.000–1.041)	0.052
DBP (/1 mmHg)	0.959 (0.939–0.980)	< 0.001	0.976 (0.946–1.006)	0.113
Urinary output(/1 ml/day)	0.999 (0.999–1.000)	0.090	1.000 (0.999–1.000)	0.255
Dialysate glucose load (/1 g/day)	1.004 (0.999–1.010)	0.138	1.007 (0.999–1.016)	0.078
Dialysate calcium load (/1 g/day)	0.703 (0.053–9.290)	0.789	1.177 (0.031–45.263)	0.930
Total Kt/V(/1)	1.011 (0.772–1.324)	0.936	1.094 (0.861–1.390)	0.461
nPCR (/1 g/d)	0.903 (0.355–2.300)	0.831	1.186 (0.312–4.514)	0.802
HB (/1 g/L)	0.974 (0.947–1.001)	0.059	0.964 (0.927–1.003)	0.067
ALB (/1 g/L)	0.844 (0.780–0.914)	< 0.001	0.867 (0.775–0.971)	0.013
Ferritin (/1 ng/ml)	1.001 (1.000–1.001)	0.118	1.000 (0.999–1.002)	0.472
Ca (/1 mmol/L)	1.461 (0.597–3.573)	0.407	1.496 (0.386–5.802)	0.560
P (/1 mmol/L)	1.261 (0.587–2.708)	0.552	2.256 (0.766–6.641)	0.140
IPTH (/1 pg/ml)	0.999 (0.997–1.000)	0.114	1.000 (0.998–1.002)	0.843
ALP (/1 U/L)	1.001 (0.992–1.009)	0.874	1.004 (0.992–1.015)	0.517
HCO3^−^ (/1 mmol/L)	0.883 (0.788–0.990)	0.033	0.902 (0.767–1.062)	0.216
TG (mmol/L)				
< 1.70	Ref.		Ref.	
≥ 1.70	1.865 (1.128–3.085)	0.015	2.041 (0.977–4.264)	0.058
LDL-C (/1 mmol/L)	1.423 (1.050–1.928)	0.023	1.710 (1.128–2.592)	0.011
Abdominal aortic calcification (presence vs. absence)	4.435 (2.486–7.910)	< 0.001	5.761 (2.345–14.154)	< 0.001

**Fig. 3:**
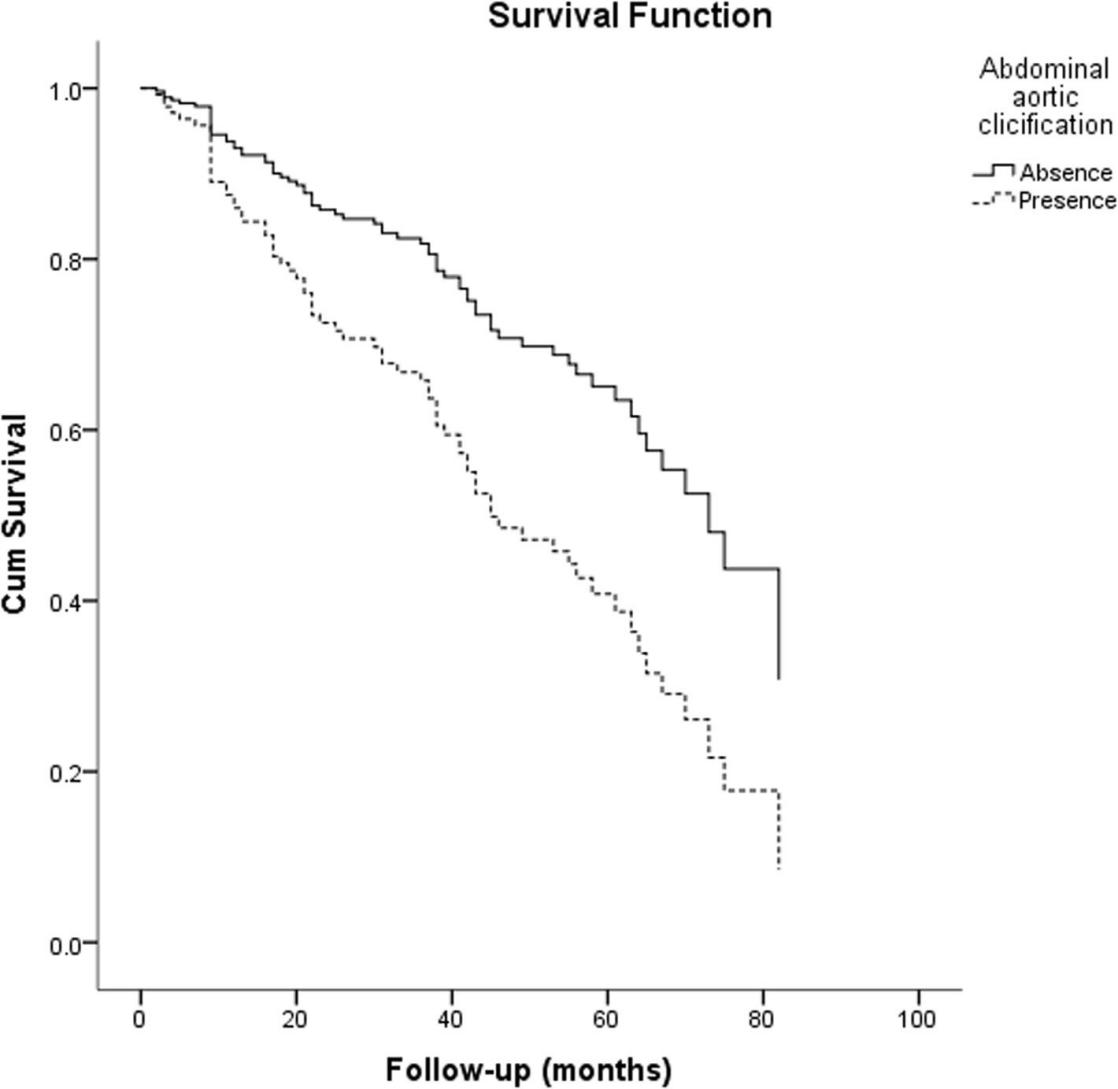
The survival curve of all-cause mortality of patients after adjusted for age, diabetes, BMI, hypertension, DBP, ALB, TG, HCO3- and LDL-C. Patients with abdominal aortic calcification showed significantly greater all-cause death than those without; Multivariate Cox Proportional Hazards Analysis, *P* = 0.029

**Fig. 4:**
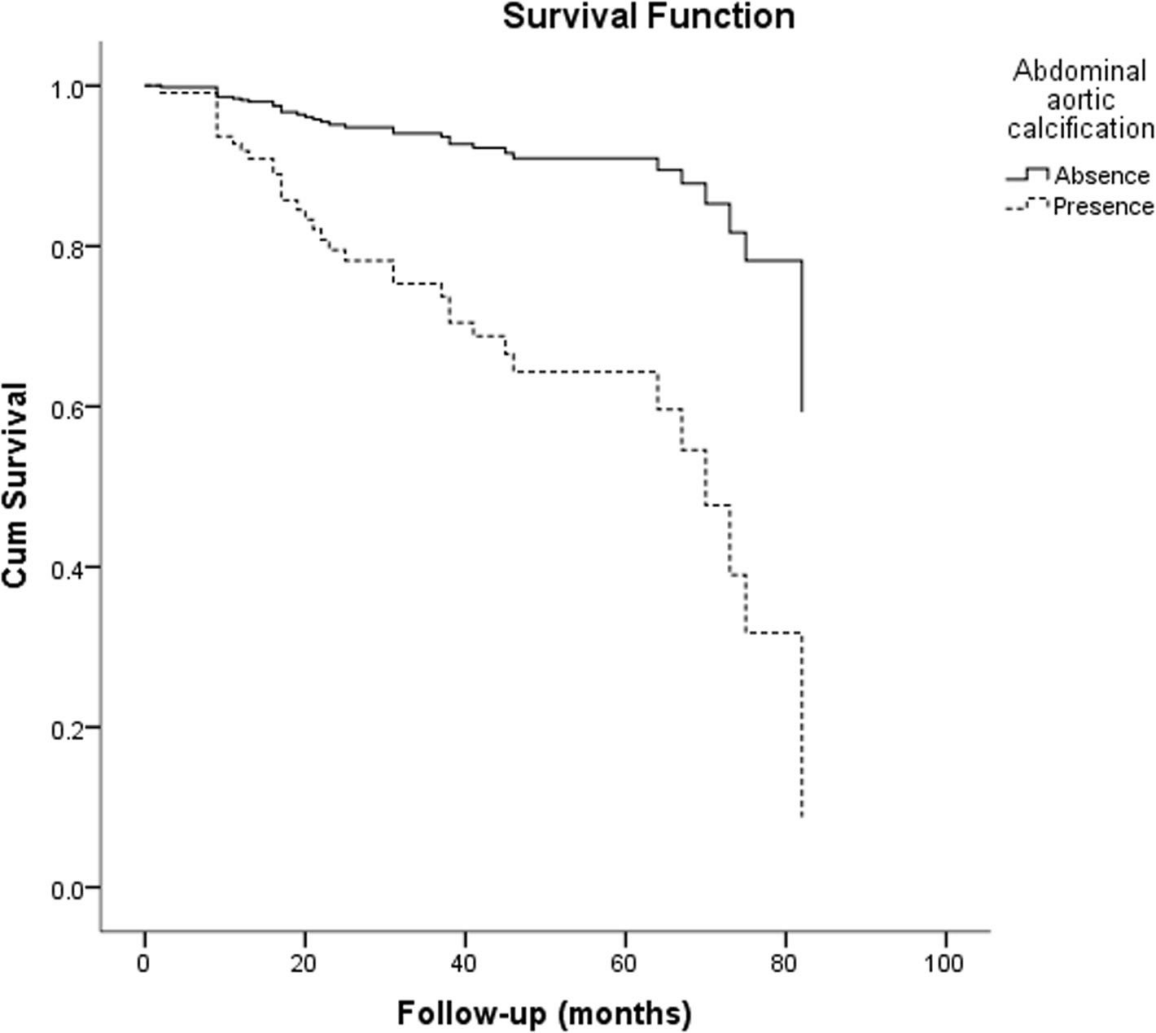
The survival curve of cardiovascular mortality of patients after adjusted for age, diabetes, cerebrovascular disease, ALB and LDL-C. Patients with abdominal aortic calcification showed significantly greater cardiovascular death than those without; Multivariate Cox Proportional Hazards Analysis, *P* = 0.001

Variables that showed *P* < 0.05 on univariate COX regression analysis were entered as possible factors in the multivariate COX regression model. The presence of AAC was a significant factor associated with all-cause mortality (HR = 2.089, 95% CI: 1.089–4.042, *P* = 0.029, Table 3) in addition to older age and diabetes. Similarly, the presence of AAC was an independent predictor of CV mortality (HR = 4.660, 95% CI: 1.852–11.725, *P* = 0.001, Table 3) in addition to previous history of cerebrovascular disease, diabetes. The all-cause mortality and CV mortality of the patients with AAC were significantly higher than those without AAC (Figs. 3 and 4).

**Table 3:** Multivariate Cox proportional hazards analysis of factors associated with all-cause and cardiovascular mortality in peritoneal dialysis patients

Variable	All-cause mortality		Cardiovascular mortality	
	HR (95% CI)	*P*	HR (95% CI)	*P*
Age (years)				
< 65	Ref.		Ref.	
≥ 65	3.264 (1.802–5.912)	< 0.001	1.397 (0.602–3.245)	0.436
Cerebrovascular disease (yes vs. no)	–	–	2.843 (1.258–6.423)	0.012
Hypertension (yes vs. no)	1.503 (0.693–3.258)	0.302	–	–
Diabetes (yes vs. no)	1.868 (1.125–3.101)	0.016	2.802 (1.310–5.996)	0.008
BMI (kg/m^2^)				
< 18.50	Ref.			
18.50–23.99	2.347 (0.828–6.657)	0.109	–	–
≥ 24.00	2.497 (0.866–7.196)	0.090	–	–
DBP (/1 mmHg)	0.991 (0.963–1.020)	0.556	–	–
ALB (/1 g/L)	0.931 (0.854–1.015)	0.107	0.929 (0.819–1.052)	0.250
HCO3^−^ (/1 mmol/L)	1.011 (0.905–1.130)	0.841	–	–
TG (mmol/L)				
< 1.70	Ref.			
≥ 1.70	1.287 (0.741–2.237)	0.371	–	–
LDL-C (/1 mmol/L)	1.145 (0.809–1.619)	0.445	1.429 (0.916–2.230)	0.116
Abdominal aortic calcification (presence vs. absence)	2.089 (1.089–4.042)	0.029	4.660 (1.852–11.725)	0.001

### Other vascular calcifications

The occurrence of vascular calcification in various sites is shown in Table 4. A total of 108 (72.0%) patients had VC, and the prevalence of AAC was the highest (60.7%), followed by femoral artery and iliac artery. The prevalence of calcification of finger arteries was the lowest, with only 13 (8.7%). According to the VC score, 42 patients (28.0%) were in the group without calcification, 61 (40.7%) in the group of mild calcification, 30 (20.0%) in the group of moderate calcification, and 17 (11.3%) in the group of severe calcification (Table 4).

**Table 4:** Vascular calcification in different sites of PD patients (*n* = 150)

	Presence, n(%)	Absence, n(%)
Different sites of vascular calcification	108 (72.00)	42 (28.00)
Abdominal aortic calcification	91 (60.67)	59 (39.33)
Iliac artery calcification	37 (24.67)	113 (75.33)
Femoral artery calcification	54 (36.00)	96 (64.00)
Radial artery calcification	35 (23.33)	115 (76.67)
Finger arteries calcification	13 (8.67)	137 (91.33)
Vascular calcification scores		
No calcification	42 (28.00)	
Mild calcification (1–3 points)	61 (40.67)	
Moderate calcification (4-6points)	30 (20.00)	
Severe calcification (7-10points)	17 (11.33)	

Univariate COX regression analysis was performed for the presence or absence of calcification of iliac artery, femoral artery, radial artery, and finger arteries. It was found that calcification of iliac artery and femoral artery were predictors of all-cause mortality in patients, and calcification of iliac artery and femoral artery were predictors of CV mortality. In multivariate COX regression analysis, after adjusted for age, diabetes, DBP, ALB, TG, HCO3^−^ and LDL-C, only femoral artery calcification was an independent predictor of all-cause mortality in patients (Table 5); after adjusted for age, diabetes, ALB, LDL-C, cerebrovascular disease, only femoral artery calcification can independently predict the increasing risk of CV mortality in PD patients (Table 6).

**Table 5:** Univariate and multivariate Cox proportional hazards analysis of the predictive value of calcification in different vascular for all-cause mortality

Variable	Univariate		Multivariate^a^	
	HR (95% CI)	*P*	HR (95% CI)	*P*
Iliac artery calcification	2.473 (1.529–4.001)	< 0.001	1.467 (0.875–2.459)	0.147
Femoral artery calcification	3.193 (1.981–5.146)	< 0.001	2.215 (1.355–3.620)	0.002
Radial artery calcification	1.542 (0.917–2.591)	0.102	–	–
Finger arteries calcification	1.092 (0.439–2.717)	0.849	–	–
Calcification groups				
No calcification	Ref.		Ref.	
Mild	5.462 (2.286–13.054)	< 0.001	2.905 (1.154–7.316)	0.024
Moderate	10.444 (4.190–26.035)	< 0.001	4.931 (1.860–13.069)	< 0.001
Severe	7.324 (2.583–20.766)	< 0.001	5.680 (1.979–16.301)	< 0.001

^a^adjusted for age, diabetes, BMI, hypertension, DBP, ALB, TG, HCO3^−^ and LDL-C

**Table 6:** Univariate and multivariate Cox proportional hazards analysis of the predictive value of calcification in different vascular for cardiovascular mortality

Variable	Univariate		Multivariate^a^	
	HR (95% CI)	*P*	HR (95% CI)	*P*
Iliac artery calcification	3.441 (1.757–6.739)	< 0.001	1.877 (0.906–3.892)	0.090
Femoral artery calcification	4.070 (1.999–8.288)	< 0.001	3.651 (1.760–7.573)	0.001
Radial artery calcification	1.881 (0.914–3.872)	0.086	–	–
Finger arteries calcification	1.412 (0.430–4.640)	0.570	–	–
Calcification groups				
No calcification	Ref.		Ref.	
Mild	4.261 (1.204–15.079)	0.025	2.568 (0.684–9.644)	0.162
Moderate	10.923 (2.982–40.011)	< 0.001	8.211 (2.088–32.299)	0.003
Severe	12.622 (3.162–50.392)	< 0.001	10.746 (2.711–42.593)	0.001

^a^adjusted for age, diabetes, cerebrovascular disease, ALB and LDL-C

**Fig. 5:**
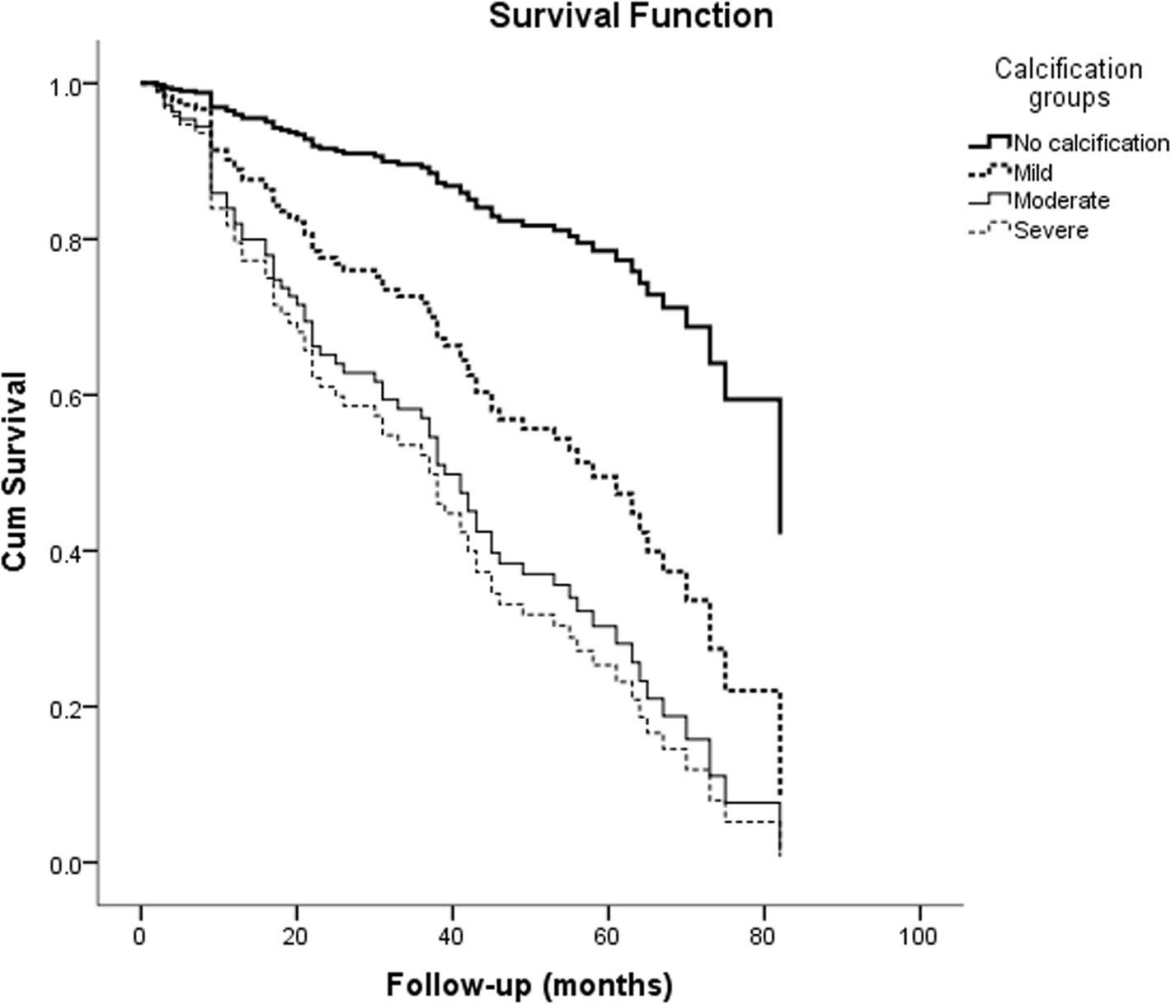
The survival curve of all-cause mortality of patients in different groups after adjusted for age, diabetes, BMI, hypertension, DBP, ALB, TG, HCO3- and LDL-C

**Fig. 6:**
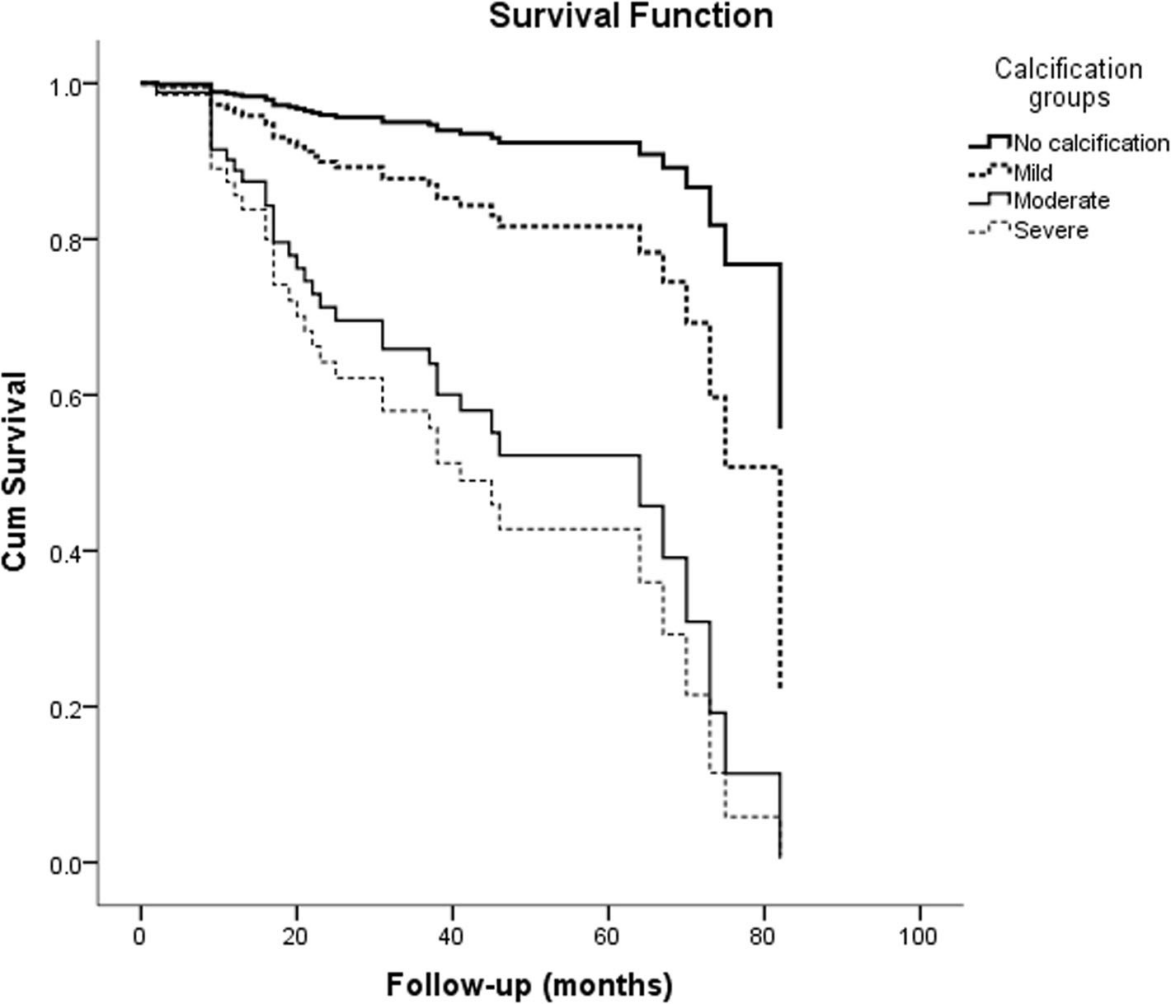
The survival curve of cardiovascular mortality of patients in different groups after adjusted for age, diabetes, cerebrovascular disease, ALB and LDL-C

The effects of different severity of VC on patients’ prognosis were estimated by univariate and multivariate COX regression analysis. In multivariate COX regression analysis, after adjusted for variables, mild calcification, moderate calcification and severe calcification were independent predictors of all-cause mortality in patients comparing absence of VC. The risk of all-cause mortality in patients with moderate or severe calcification was significantly higher than that in patients with mild calcification and without calcification (Table 5). In the analysis of predictors of CV mortality, moderate and severe calcification were independent predictors after adjusting for age, diabetes, ALB, LDL-C, and history of cerebrovascular disease (Table 6). The survival curves of the groups adjusted by variables were shown in Figs. 5 and 6.

## Discussion

According to our results, AAC was an independent predictor of all-cause mortality and CV mortality in PD patients. In addition to AAC, femoral artery calcification was also an independent predictor of all-cause mortality and CV mortality, while VC in other sites had no predictive effect on patients’ prognosis. And the VC scores also had a predictive effect on the prognosis of PD patients, as the total VC score increases, the risk of all-cause mortality and CV mortality increases.

The abdominal aorta is a site that is prone to atherosclerosis and calcification, and it is a good indicator of VC in patients and can predict all-cause and CV mortality according to previous studies [27–29]. Moreover, in order to observe the position of the PD catheter and the intra-abdominal condition, PD patients usually performed imaging examinations such as abdominal X-ray or abdominal CT. Using these imaging films to evaluate AAC can provide important information for the management of CVD in PD patients without any additional cost. And evaluating the presence or absence of VC or calcification scores is convenient for clinicians to perform. However, VC in other areas has received little attention and assessment of the predictive value of mortality. Femoral artery is a medium-sized artery. We found that femoral artery calcification was a good predictor of risk of all-cause mortality and CV mortality in PD patients. Moreover, it is easy to know whether or not the femoral artery is calcified by X-ray film. For patients with high risk of VC, the calcification of the abdominal aorta and femoral artery can be evaluated simultaneously, and the VC burden caused by CKD-MBD and the effect of prognosis can be more accurately understand.

Among VC in different sites, the prevalence of AAC was the highest (60.7%), and the prevalence of calcification of the iliac artery, femoral artery, radial artery and finger arteries was significantly lower than that of the abdominal aorta. Even the calcification of the small and medium arteries was mostly accompanied by calcification of large artery, there still independent existence of calcification in small and medium arteries. In the study of O’Neil et al. [22], by comparing the calcification of small arteries and arterioles in CKD patients and controls, they found that vascular smooth muscle cells maintained the normal phenotype and no apoptosis in CKD patients. And they found that media calcification in small arteries didn’t involved osteogenic transdifferentiation of vascular smooth muscle cells. These were not equal to the pathogenesis of medial calcification of other arterial types. Indeed, the susceptibilities of calcification were different in vary artery sizes and types. The different ontogenic origin of different portions of vascular smooth muscle cells maybe one of the main mechanisms [30].

VC may affect the prognosis of patients through the following aspects. First, intimal calcification may aggravate atherosclerosis, thereby increasing the risk of developing arterial occlusive disease [31, 32]. Second, VC reduces arterial elasticity and causes an increase in arterial stiffness. Decreased aortic reserve function leads to a decrease in diastolic blood flow in the coronary arteries, which ultimately leads to myocardial ischemia [33]. Third, long-term left ventricular load aggravation can cause left ventricular hypertrophy, and gradually develop into heart failure [33]. In addition to AAC, age and diabetes are traditional risk factors for increased risk of mortality in CKD patients. The cause of death in some patients in the present study was diabetic foot-related infection. A history of cerebrovascular disease, diabetes, and high level of LDL-C were independent predictors of CV mortality in patients. Patients with a history of cerebrovascular disease tend to have a significantly increased risk of cerebrovascular accidents. Diabetes and dyslipidemia accelerate the development and progression of atherosclerosis and VC, further increasing the incidence of CVD and CV events.

Previous studies have shown that high levels of serum P, Ca and iPTH were risk factors for VC. IPTH can increase the expression of cartilage matrix and increase the intracellular calcium content, thereby promoting the formation of VC [32, 34]. Meanwhile, the serum levels of Ca, P and iPTH were related to the occurrence of CVD and CV mortality in ESRD patients [35, 36]. However, the results of the present study showed that serum levels of Ca, P, and iPTH were not significantly different between the two groups with or without AAC, and they were not associated with prognosis. The possible reasons were: our center has consistently adhered to continuous quality improvement (CQI) for Ca and P metabolic disorders, so the laboratory indices of most patients are within the target range. The average serum Ca of the selected patients was 2.32 mmol/L, P 1.49 mmol/L, iPTH 140 pg/ml, which minimized the influence of Ca and P metabolic disorders on patients.

There were also some limitations in this study. First, the study was designed as a retrospective cohort study, and there were some differences in baseline characteristics between groups at the time of enrollment. Even if the relevant variables were corrected during the statistical analysis process, the effect of confounding factors was inevitable. Second, the number of sample size was relatively small. But, the present study can objectively estimate the predictive effect of VC of various sites on the prognosis of PD patients, and further guide the clinical management and treatment of CKD-MBD.

## Conclusions

In conclusion, we evaluated VC in different sites of PD patients by a retrospective cohort study. The results showed that the prevalence of AAC was pretty high (60.7%), and AAC was an independent predictor of all-cause mortality and CV mortality in PD patients. In other sites of VC, femoral artery calcification can predict the increasing risk of all-cause mortality and CV mortality in patients, as the calcification in iliac, radial, and finger arteries were not associated with the prognosis of PD patients. And the VC score had a predictive effect on the mortality of PD patients. Therefore, in daily clinical work, excessive imaging examinations of VC should be avoided to reduce unnecessary X-ray exposure and medical resources waste. The imaging data of PD patients such as abdominal radiographs and CT scans should be fully utilized to evaluate the condition of AAC. If necessary, the risk of CVD and mortality can be assessed based on the calcification of the abdominal aorta and femoral artery.

## Data Availability

The datasets used during the current study are available from the corresponding author on reasonable request.

## Abbreviations

AAC: Abdominal aortic calcification
ALB: Albumin
ALP: Alkaline phosphatase
BP: Blood pressure
Ca: Calcium
CI: Confidence intervals
CKD-MBD: Chronic kidney disease-mineral and bone disorder
CO2CP: Carbon dioxide combining power
CVD: Cardiovascular disease
EBCT: Electron beam computed tomography
ESRD: End stage renal disease
HB: Hemoglobin
HR: Hazard ratio
iPTH: Serum intact parathyroid hormone
KDIGO: Kidney Disease: Improving Global Outcomes
Kt/V: Total urea clearance
LDL-C: Low-density lipoprotein cholesterol
MSCT: Multislice computed tomography
P: Phosphate
PD: Peritoneal dialysis
TG: Triglycerides
VC: Vascular calcification
